# Self-Healing Thermal-Reversible Low-Temperature Polyurethane Powder Coating Based on Diels–Alder Reaction

**DOI:** 10.3390/ma17143555

**Published:** 2024-07-18

**Authors:** Katarzyna Pojnar, Barbara Pilch-Pitera, Shahla Ataei, Patrycja Gazdowicz, Beata Mossety-Leszczak, Beata Grabowska, Artur Bobrowski

**Affiliations:** 1 Doctoral School of Engineering and Technical Sciences, Rzeszow University of Technology, ul. Powstańców Warszawy 12, 35-959 Rzeszów, Poland; 2 Faculty of Chemistry, Rzeszow University of Technology, ul. Powstańców Warszawy 6, 35-959 Rzeszów, Poland; 163711@stud.prz.edu.pl (P.G.); mossety@prz.edu.pl (B.M.-L.); 3 Harsin Chemical Homa Company, Garmsar 3593147487, Iran; sh.ataei.k@gmail.com; 4 Faculty of Foundry Engineering, AGH University of Science and Technology, ul. Reymonta 23, 30-059 Kraków, Poland; beata.grabowska@agh.edu.pl (B.G.); arturb@agh.edu.pl (A.B.)

**Keywords:** low-temperature-curing powder coatings, reversible Diels–Alder reaction, self-healing

## Abstract

This work focused on obtaining a low-temperature powder coating characterized by self-healing properties. To achieve this, acrylic resin, blocked polyisocyanates (bPICs) with 1,2,4-triazole, and unsaturated commercial resin were used. The synthesis of bPICs with triazole enabled the low-temperature curing and reversible Diels–Alder (DA) reaction at 160 °C. The chemical structure of bPICs was confirmed using ^1^H-NMR. The occurrence of the DA and retro-DA (rDA) reactions in the crosslinked polymer, at temperatures of 60–85 °C and 90–130 °C, respectively, was confirmed using Differential Scanning Calorimetry (DSC), Thermogravimetric Analysis (TGA), and FT-IR spectroscopy. The self-healing properties of the powder coating were examined using polarized optical microscopy. Additionally, the occurrence of the DA and rDA reactions between triazole and unsaturated polyester resin was investigated through repeated self-healing tests.

## 1. Introduction

The growing market demand for increasingly eco-friendly products and coating processes necessitates the exploration of innovative solutions. Currently, the most environmentally sustainable options available are powder coatings and paints, which do not emit volatile organic compounds (VOCs) like solvent-based products and do not contain biocides to protect them from microorganism growth in packaging, as is the case with water-based products [1,2]. For these reasons, powder coatings play a crucial role in the chemical industry. The “5E” standards, such as efficiency, economy, energy savings, environmental compliance, and excellence of finish, of powder coatings are fulfilled [3]. A significant drawback of standard high-temperature powder coating systems is the high energy consumption during the curing process, which typically occurs at temperatures ranging from 180 to 220 °C [4]. To address this issue and reduce energy usage, ongoing research aims to lower the curing temperature. Innovations in this area include the development of special crosslinking agents, resins, and additives that enable curing at temperatures below 160 °C or through UV radiation. [5,6].

Acrylic resins allow for the development of low-temperature powder coating systems and UV-cured coatings [7,8]. Additionally, they possess excellent properties, including superior weatherability and high chemical, thermal, and mechanical resistance. This is demonstrated by their hydrophobic properties, color durability, and high resistance to scratches, sand abrasion, and damage caused by stones [9]. Due to the presence of functional groups within acrylic resin, powder coatings can be divided into three categories containing carboxyl, epoxy, and hydroxyl groups [10]. Acrylic resin containing carboxyl groups, due to issues with flexibility, is most commonly found in a hybrid system. Okada et al. described an acrylic/polyester hybrid powder coating [11]. Polyester powder coatings, featuring both -COOH and -OH functionality, offer advantages such as good appearance and favorable mechanical properties like toughness and flexibility. However, their drawback lies in insufficient weather durability [11]. Acrylic resins containing epoxy groups are characterized by good mechanical properties such as rigidity, toughness, and adhesion [12]. Additionally, they enable UV curing through cationic polymerization [8]. Acrylic resins containing hydroxyl groups, in reaction with blocked polyisocyanates (bPICs), can enable curing temperatures below 160 °C (low-temperature systems). Additionally, compared to acrylic resins containing carboxyl or epoxy groups, they exhibit the best flexibility properties [13]. This is one of the reasons for choosing them for use in this research.

In the literature, the known blocking agents are imidazoles, amides, oximes, phenols, triazoles, imides, pyrazoles, hydroxyamic acid esters, and active methylene compounds [14,15]. Czachor-Jadacka et al. described crosslinking agents based on blocked polyisocyanates that induce curing at reduced temperatures. An example is methyl ethyl ketoxime (MEKO)-blocked PICs or butanone oxime, which, compared to ε-caprolactam-blocked PICs, undergo deblocking at temperatures below 160 °C rather than 180 °C [16].

The Diels–Alder (DA) reaction, as a non-autonomic process, is one of the most well-known and common reactions used in the structure design of self-healing polymers. Due to the thermal reversibility of the DA bonds, the microcracks are healed as many times as needed by undergoing a cooling/heating cycle, followed by the reforming of the DA bonds [17]. The reaction involves a [4 + 2] cycloaddition between an electron-rich conjugated diene and a substituted alkene (a poor electron dienophile). This forms strong dynamic covalent bonds, resulting in the formation of a substituted cyclohexane as the DA adduct [18]. Due to the low energy consumption for the formation of this cyclohexane ring, it also provides the possibility of forming and functionalizing different molecules.

In summary, the healing process through crosslink formation includes two steps:

(1) Upon the cracks being formed on a microscopic scale, healing may be achieved by forming a network through the discharge of crosslinks formed between the diene and dienophile in that place. As the temperature increases (around 120–160 °C), the equilibrium reaction shifts from the side of DA bonds formed toward the breaking of these bonds, which leads to an increase in molecular mobility and a higher concentration of active diene and dienophile groups. At a certain temperature, the contact between the cracked surfaces is facilitated by decreasing the crosslinking density, which in turn helps to close the microscopic cavities.
(2) The sample is annealed to the temperature required for the DA reaction and retrieval of bonds to form a DA moiety. Since the crosslinks are formed through an equilibrium reaction between a diene and dienophile and the formation of a DA linkage is exothermic, upon the temperature decrease (50–70 °C), the equilibrium shifts to the reversed direction, i.e., the more bonded state, and as a result, dynamic covalent crosslinks are formed at the crack interface during the [4 + 2] cycloaddition DA reaction. Thus, this process can be repeated at a suitable and effective temperature until the crack is filled [19,20].

The DA reaction has been used in many works to obtain the self-healing effect of coatings, including polyurethane coatings in the work of Farshchi N. et al. [21]. In order to achieve the self-healing effect, an additional self-healing agent was introduced containing furfuryl alcohol and maleic anhydride adduct. The innovation of this study is the use of a blocking agent to create a self-healing adduct, which will additionally eliminate its emission into the environment.

The aim of this study was to examine the effect of the addition of unsaturated polyester resin to the low-temperature-curing polyurethane powder coating formulation on the self-healing properties. The key feature of this coating was the ability to cure at a low temperature. This was achieved by incorporating a triazole blocked polyisocyanates and an acrylic resin containing hydroxyl groups. To start the curing process, the unblocking reaction was performed at 110–130 °C. During the curing process, the unblocked -NCO groups reacted with the -OH groups of the acrylic resin, resulting in the formation of low-temperature-curing powder coatings. In addition, at the same time, the triazole released from the unblocking reaction was able to react with the commercial unsaturated polyester resin, which allowed the DA reaction to occur. Various analytical techniques such as DSC, FTIR, polarized optical microscopy, repeated self-healing test, and TGA were used to evaluate the effectiveness of low-temperature curing and the DA reaction. The obtained results confirmed that the new powder coatings exhibit a low VOC emission, a self-healing effect, and better physical–mechanical properties.

## 2. Experimental Section

### 2.1. Reagents

Raw materials employed in the synthesis of the acrylic resin: 2-hydroxyethyl methacrylate (HEMA) (Merck, Darmstadt, Germany), methyl methacrylate (MMA) (Sigma Aldrich, Darmstadt, Germany), *n*-butyl acrylate (BA) (Sigma Aldrich, Darmstadt, Germany), and azobisisobutyronitrile (AIBN; Sigma Aldrich, Darmstadt, Germany).

Raw materials utilized in the synthesis of blocked polyisocyanate (bPIC): isophorone diisocyanate (IPDI; Evonik Industries, Essen, Germany), dibutyltin dilaurate (Sigma Aldrich, Darmstadt, Germany), glycerin (Chempur, Piekary Śląskie, Poland), α,ω bis(hydroxyethyleneoxypropylene)polydimethylsiloxane (KF6000) with LOH = 120 mg KOH/g (Shin-Etsu, Tokio, Japan), and 1,2,4-triazole as a blocking agent (Sigma Aldrich, Darmstadt, Germany).

Commercial unsaturated resins: UVECOAT 3003 (Meth)acrylated epoxy/polyester resin (Allnex, Frankfurt, Germany).

### 2.2. Synthesis of Acrylic Resin

The acrylic resin was synthesized via bulk free radical polymerization, employing the following monomers: 2-hydroxyethyl methacrylate (HEMA), methyl methacrylate (MMA), *n*-butyl acrylate (BA), and 1.7% azobisisobutyronitrile (AIBN). Therefore, the acrylic resin was named according to the names of the monomers used, HEMA, MMA, and BA, in a molar ratio of 1:5:2, e.g., HEMA/5MMA/2BA [22].

### 2.3. Synthesis of Blocked Polyisocyanates (bPICs)

IPDI and dibutyltin dilaurate catalyst (0.1 wt% based on diisocyanate) were introduced into a three-necked flask. Additionally, a reflux condenser, thermometer, glass stirrer, nitrogen inlet, and dropping funnel were used. Simultaneously, glycerin (Chempur, Piekary Śląskie, Poland) and KF-6000 (Shin-Etsu, Tokio, Japan) were mixed in a beaker before being slowly added to the diisocyanate in the flask. The reaction mixture was then maintained at 90 °C, stirred, and refluxed for a duration of 1.5 h. Triazole, serving as a blocking agent, was added to the obtained polyisocyanate at a 1:1 ratio of -NCO to -NH, for 1 h at 115 °C under stirring. The names of the samples were designated using the first letters of the compound, for example, IGKF/T indicates a blocked polyisocyanate synthesized from IPDI (“I”), glycerin (“G”), and KF-6000 (“KF”) and blocked with triazole (“T”).

### 2.4. Preparing Self-Healing Powder Coating Composition and Coatings

The self-healing powder coating composition consisted of an acrylic resin HEMA/5MMA/BA, bPIC, and an appropriate selected commercial unsaturated resin, UVECOAT 3003. The hydroxyl group contained in acrylic resin in the reaction with the deblocked polyisocyanate was used in the curing process of powder coatings. A co-rotating twin-screw mini extruder EHP 2 × 12 Sline from Zamak (Cracow, Poland) was used to homogenize the mixture. The temperature settings of the extruder were as follows: Zone I—75 °C; Zone II—90 °C; Zone III—100 °C; and adapter—110 °C. In the next steps, the composition was cooled, pulverized, and passed through a 100 μm sieve. The resulting powder coatings were applied to the Q-panels using the CORONA electrostatic method with a WAGNER PEM X1 gun (Wagner, Alstatten, Switzerland). Subsequently, the powder coatings were cured at 160 °C for 15 min. After the curing process, the powder coatings were named, for example, L_HEMA/5MMA/2BA/IGKF/T/UVECOAT 3003 indicates a coating made from the resin HEMA/5MMA/2BA (qualitative composition), IGKF/T (bPIC), and UVECOAT 3003 (chosen commercial resin) (Table 1).

## 3. Characteristics of the Methods Used

### 3.1. Nuclear Magnetic Resonance Spectroscopy (NMR)

The Bruker Avance II 500 MHz spectrometer (Bruker BioSpin, Rheinstetten, Germany), equipped with a 5 mm nitrogen-cooled dual (BB-1H) cryoprobe, was used for the experiments. Chemical shifts are reported in parts per million (ppm) relative to tetramethylsilane (TMS) as the internal standard, with deuterated chloroform (CDCl3) used as the solvent. Data analysis was conducted using NMR Topspin 2.1 pl 8 software (Bruker BioSpin, Rheinstetten, Germany).

### 3.2. Differential Scanning Calorimetry (DSC)

The thermal properties of the powder compositions were analyzed using a Mettler Toledo type 822e differential scanning calorimeter (DSC) (Mettler Toledo, Columbus, OH, USA), operated with Stare System software 16.20. Heating was conducted at a rate of 10 °C/min. Aluminum crucibles containing samples weighing 0.015 g were placed into the measurement chamber. Measurements were performed in a nitrogen atmosphere at a flow rate of 60 cm^3^/min, covering a temperature range from 0 to 160 °C.

### 3.3. FT-IR Measurements

The IR spectra were acquired using a Thermo Scientific Nicolet 6700 FT-IR spectrophotometer (ThermoFisher Scientific, Waltham, MA, USA) equipped with a helium–neon (HeNe) laser. Spectra were recorded over the range of 700–4000 cm^−1^ with a resolution of 4 cm^−1^. The data are presented as transmittance (%) versus wavenumber ν (cm^−1^).

### 3.4. Polarized Optical Microscopy

A VHX-7000 polarized optical microscope with an EA-300 laser by Keyence (Osaka, Japan) was employed for this study. The sample of cured and cracked powder coatings was positioned on a heating microscope table. The heating was conducted at a rate of around 20 °C/min, and the powder coating self-healing process was evaluated.

### 3.5. Thermogravimetric Analysis (TGA)

The TGA and DTG were performed using a Mettler Toledo TGA/DSC instrument (Greifensee, Switzerland) equipped with Stare System software. TGA experiments were conducted under a nitrogen atmosphere, ranging from 25 to 600 °C at a heating rate of 10 °C/min. The experimental parameters were as follows: sample weight of approximately 5 mg, gas flow rate of 50 cm^3^/min, and an open alumina pan with a volume of 150 μL.

### 3.6. Polymerization Test

The test involved rubbing the powder coatings with a swab soaked in MEK (methyl ethyl ketone) lightly back and forth 30 times in each direction. The evaluation was conducted according to the guidelines outlined in the “Technical requirements of the QUALICOAT quality label” [23]. Assessment took place 30 min after rubbing, and coatings were categorized based on the following criteria:

Coating appears matte and soft;
Coating appears matte and can be scratched with a nail;
Slight gloss reduction;
No noticeable changes.

### 3.7. Flow Test

In order to conduct the flow test, the PN-EN ISO 8130-11 standard was used [24]. The test plates were 6.6 mm deep, and 0.4 g of powder coatings was added. The test plates with the powder coating samples were then placed in an oven at a temperature of 160 °C for 20 min, positioned at a 60° angle from the horizontal. Finally, the distance between the bottom edge of the recess and the furthest point reached by the molten powder coating was evaluated.

### 3.8. Roughness

Roughness values were evaluated using a Mar Surf PSI profilometer (Göttingen, Germany) in accordance with PN-EN ISO 12085 [25]. The R_a_ parameter, which represents the arithmetic mean of the roughness profile deviations from the baseline, and the R_z_ parameter, which represents the arithmetic mean of the 5 highest profile peaks minus the arithmetic mean of the 5 lowest profile valleys, were measured [25]. The measurement was conducted automatically by moving the needle along the surface of the coating.

### 3.9. Thickness and Gloss

A micro-TRI-gloss tester from BYK-Gardner GmbH (Geretsried, Germany) was used in accordance with PN-EN ISO 2813 for gloss measurement and PN-EN ISO 2808 for thickness measurement [26,27]. Gloss measurements were taken at angles of 20°, 60°, and 85°. The same device was used for measuring thickness. The results for these parameters were obtained by averaging the measurements from ten trials for each sample.

### 3.10. Adhesion to Steel

According to PN-EN ISO 2409, adhesion to steel was evaluated by using a multi-cut tool manufactured by Byk Gardner (Geretsried, Germany) with six cutters spaced 2 mm apart [28]. In order to indicate parameters, a scale from 0 to 5 was used. A score of 0 indicated the best surface adhesion, with completely smooth incision edges, while a score of 5 indicated the worst adhesion, with more than 65% of the incision network damaged.

### 3.11. Hardness

The Konig Pendulum tester from BYK-Gardner GmbH (Geretsried, Germany), in accordance with PN-EN ISO 1522, was used to determine the relative hardness of the powder coatings [29]. The relative hardness was calculated by dividing the arithmetic mean of the number of pendulum oscillations for the tested sample by the glass constant, which is 171 pendulum oscillations. Three measurements were performed for each coating.

### 3.12. Scratch Resistance

The Clemen Tester from Elcometer (Manchester, UK) was used to check the scratch resistance of the powder coatings, according to PN-EN ISO 1518 [30]. The sample was positioned facing upwards. The device, without any load applied, was placed on the coating, and the test panel was moved outward at a speed of 30 mm/s. The scratch resistance was determined as the lowest load applied to the tool at which a scratch appeared on the cured coating.

### 3.13. Cupping

The cupping properties were determined according to the PN-EN ISO 1520 standard [31]. The results were obtained using a manual SP4300 tester by TQC (Capelle aan den Ijssel Miasto, Holandia, The Netherlands). The spherical drawing punch was used to indent a clamped sheet until the coating cracked. The point of crack initiation was then recorded. To ensure reliability, three measurements were performed on the same cured coating.

### 3.14. Water Contact Angle (WCA)

The water contact angle (WCA) was measured in accordance with PN-EN ISO 19403-6:2020-08 using an optical goniometer OCA15 EC from DataPhysics (Filderstadt, Germany), which was equipped with a digital camera [32]. The final result was determined by averaging multiple measurements, ensuring a comprehensive representation of the coating’s water contact angle.

## 4. Results and Discussion

In order to develop low-temperature and self-healing powder coatings, acrylic resin containing a hydroxyl group (-OH), blocked polyisocyanates (bPICs), commercial unsaturated resin (UVECOAT 3003), and additives were used. The first step of this research consisted of synthesizing and characterizing the acrylic resin and bPIC. Then, the isocyanate groups were unblocked at a temperature of 160 °C for 15 min. Therefore, the obtained powder coatings are classified as low-temperature systems. Subsequently, the focus shifted to examining the reversibility of the DA reaction, followed by the evaluation of the performance properties of the powder coatings.

### 4.1. Characterization of Acrylic Resin and bPICs

#### 4.1.1. Acrylic Resin

2-hydroxyethyl methacrylate (HEMA) was used as the main component in the production of acrylic resin due to its hydroxyl group, which facilitates the crosslinking process with polyisocyanates. The addition of *n*-butyl acrylate (BA) in the mixture enhances the flexibility characteristics of powder coatings, while methyl methacrylate (MMA) exhibits contrasting properties to BA. The stiffness and thermal stability of the acrylic resin are enhanced by the use of MMA. The hydroxyl number value of acrylic resin was of LOH = 40 mg KOH/g. The number-average molecular mass (M_n_) was 7870 Da, and the dispersity index was 1.95. The viscosity (30.2 Pa*s, cone 6 at a temperature of 140 °C) and the temperature glass transition (T_g_ = 40.4 °C) were also determined. The acrylic resin used in this study was thoroughly characterized in a previous study [22].

#### 4.1.2. Blocked Polyisocyanates (bPICs)

**Figure 1:**
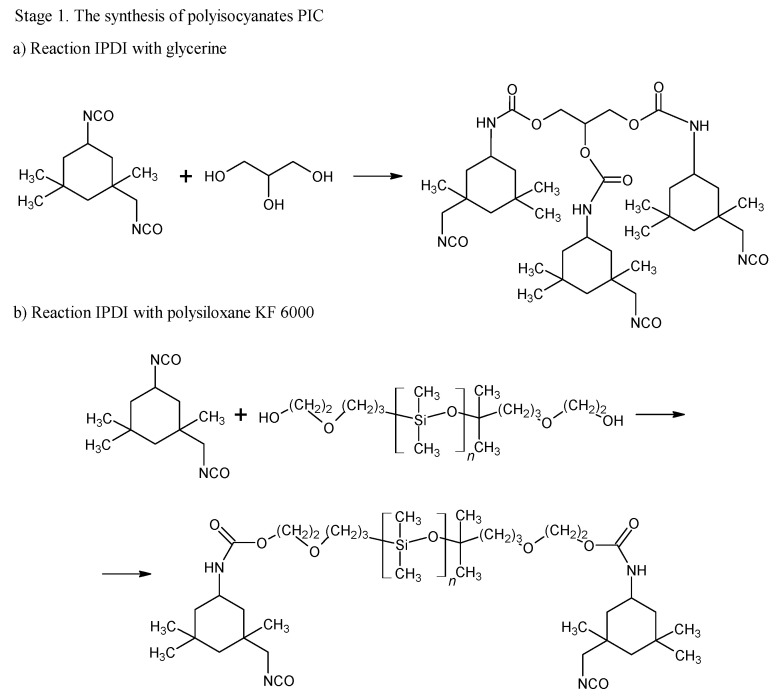

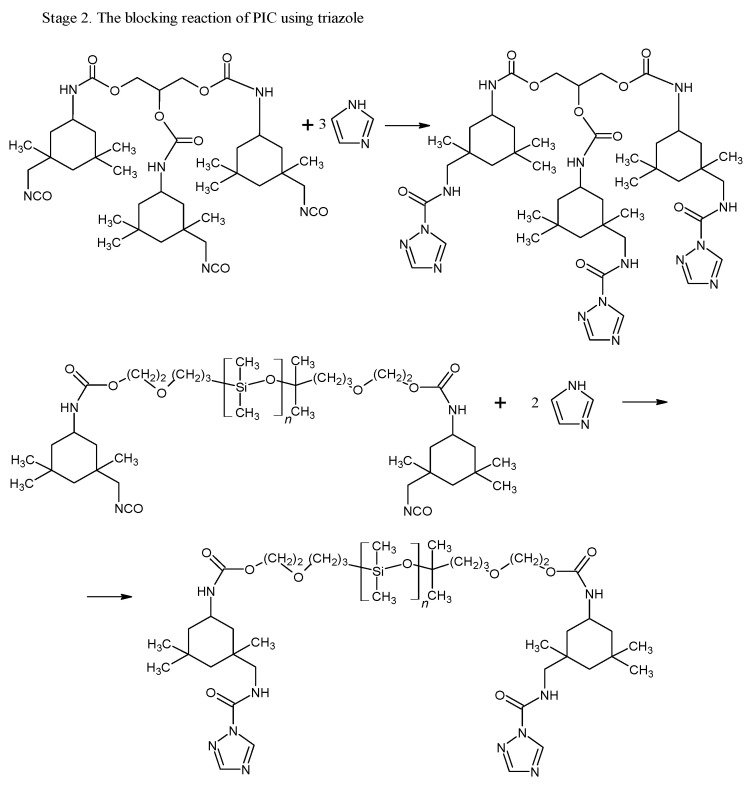
Scheme of reaction of bPIC’s.

The blocking of polyisocyanates were performed using 1,2,4-triazole, a compound capable of undergoing a Diels–Alder (DA) reaction with unsaturated polyester resin. The blocked polyisocyanates included cycloaliphatic isophorone diisocyanate (IPDI), glycerol, polysiloxane KF-6000, and 1,2,4-triazole (Figure 1). Powder coatings based on IPDI exhibit a lower tendency to yellow under light exposure compared to those made with aromatic diisocyanates, making them suitable for outdoor applications with direct exposure to weather conditions. Glycerol was used to enhance the functionality of polyisocyanates, while polysiloxane KF-6000 acted as a modifier to improve the physicochemical properties of the powder coatings [33].

**Figure 2:**
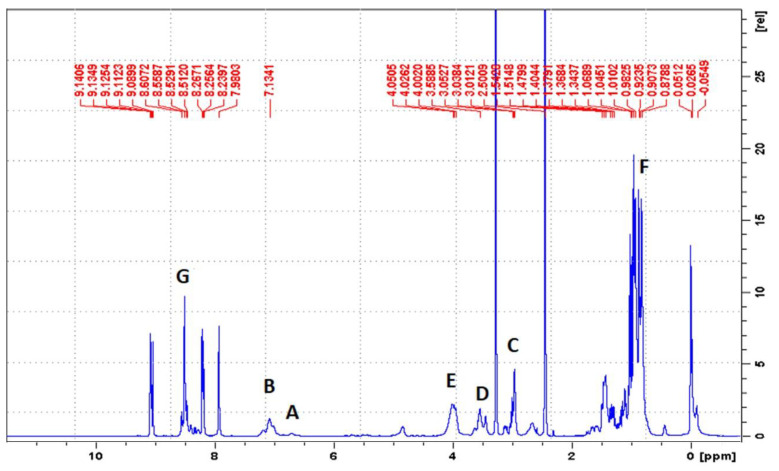
^1^H-NMR spectrum of IGKF/T.

By using ^1^H-NMR, the chemical structure of bPICs was confirmed (Figure 2). Peaks observed at 6.78 and at 7.26 ppm (labeled as “A” and “B”) indicated the presence of protons from the urethane groups, formed through the interaction between polysiloxane KF-6000 or glycerin and the -NCO groups of IPDI [34]. The double signals arising from the urethane groups reflect the ability of both the primary (at 6.78 ppm) and secondary (at 7.26 ppm) isocyanate groups in IPDI to participate in their formation. The hydrogen atoms of the methylene group adjacent to the urethane bond formed by the primary -NCO group of IPDI appear at 3.16–3.23 ppm (designated as “C”). The signal at 3.4–3.5 ppm (assigned as “D”) originated from the proton on a cycloaliphatic carbon adjacent to a urethane bond formed by the secondary -NCO group of IPDI. In this study, based on the ^1^H-NMR spectrum of blocked PICs, we were unable to determine which -NCO group of IPDI reacted first. The primary isocyanate group, due to its proximity to the methyl group, the cyclohexane ring, and the β-methyl substituent, is effectively shielded. At 4.15–4.19 ppm, signals (assigned as “E”) originating from the CH_2_ groups of polysiloxane or glycerin, adjacent to the urethane bonds, were detected. Signals from the methyl and methylene groups of IPDI were observed within the range of 0.9–1.2 ppm (assigned as “F”). The signal from the CH group of the 1,2,4-triazole moiety was assigned as “G” and observed at 8.21 ppm.

Additionally, the curing agent was evaluated for thermal stability (determining T_g_) and viscosity. The glass transition temperature (T_g_) of IGKF/T was found to be 39.41 °C, while the viscosity was determined to be 10.95 Pa*s (cone 4, at a temperature of 140 °C, speed 10 rpm). These parameters, similar to those of the resin, are crucial in the subsequent stages of manufacturing and storage of powder coatings.

### 4.2. Curing Process and Self-Healing Properties of Powder Coatings

The curing process and self-healing properties of powder coatings were also examined.

**Figure 3:**
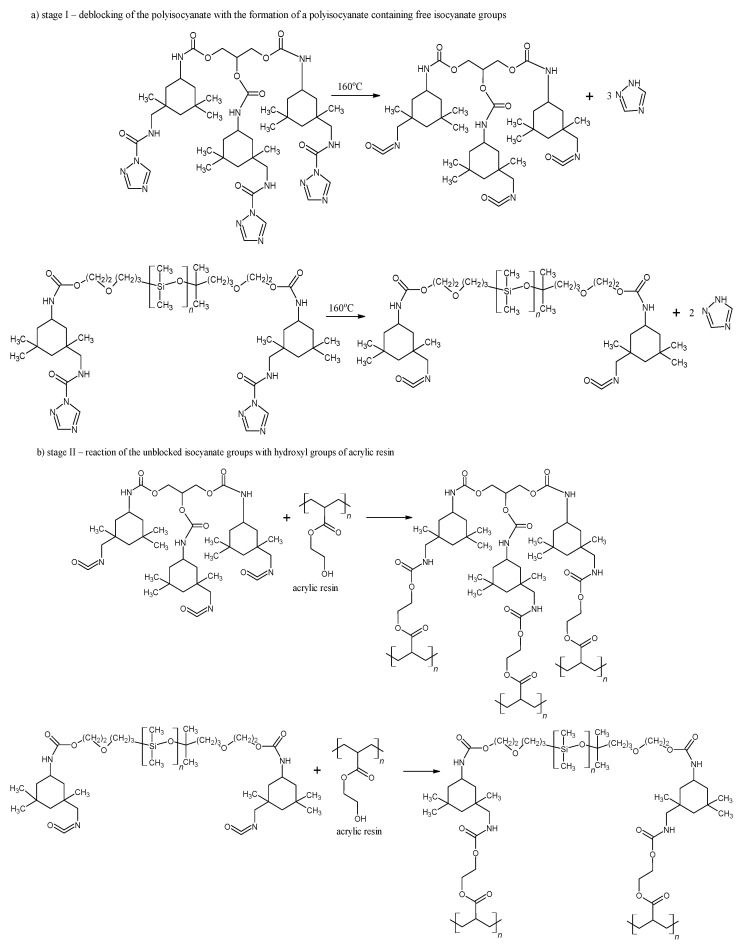
Schematic of the deblocking process for the bPICs (IGKF/T/) at 160 °C, along with the crosslinking reaction between –OH groups (originating from acrylic resin) and –NCO caused by PICs.

**Figure 4:**
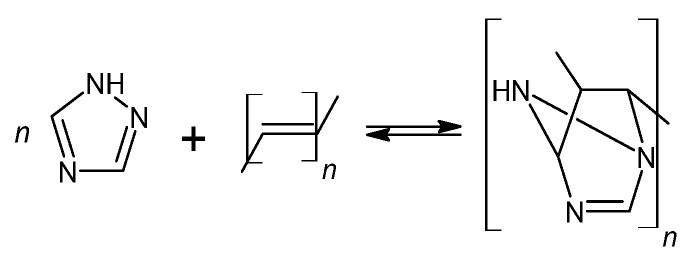
The self-healing mechanism of powder coating through a reversible DA reaction between triazole and unsaturated resin.

As shown in Figure 3, the deblocking reaction takes place at 160 °C for 15 min (Figure 3a, stage I). Following the deblocking of bPICs, the crosslinking reaction is initiated by the interaction between the free isocyanate groups in PICs and the hydroxyl groups of acrylic resin, leading to the formation of powder coatings at a reduced temperature (Figure 3b, stage II). The self-healing mechanism is then activated through the DA reaction between the triazole released from bPICs and unsaturated resin, leading to the formation of a DA moiety (Figure 4).

The course of the curing process and the reversible DA reaction of powder coatings were monitored using DSC and FT-IR techniques.

**Figure 5:**
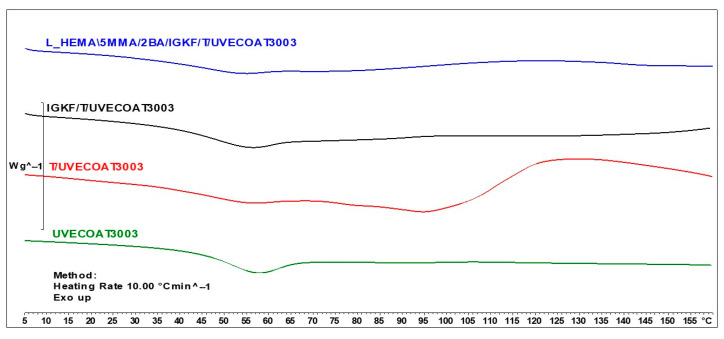
DSC curves of UVECOAT 3003, T/UVECOAT 3003, IGKF/T/UVECOAT 3003, and L_HEMA/5MMA/2BA/IGKF/T/UVECOAT 3003.

Figure 5 shows the DSC thermograms for unsaturated polyester resin UVECOAT3003, UVECOAT 3003 resin mixed with triazole T/UVECOAT3003, UVECOAT 3003 resin mixed with bPIC IGKF/T/UVECOAT 3003, and powder coating L_HEMA/5MMA/2BA/IGKF/T/UVECOAT 3003.

At the beginning of all thermograms in the range between 40 °C and 70 °C, an endothermic peak appears, indicating the transition of the resins and powder coating components to a flexible state, accompanied by enthalpy relaxation. In the thermogram of the UVECOAT 3003 resin, apart from the glass transition, no further changes are observed under the influence of controlled heating.

In the case of the sample containing UVECOAT 3003 resin and 1,2,4-triazole (T/UVECOAT3003), in addition to the glass transition, a broad exothermic peak in the range of 60–85 °C is visible, confirming the course of the DA reaction between triazole and unsaturated double bonds of UVECOAT 3003 resin. Then, an endothermic peak appears on this thermogram in the range of 90–130 °C, confirming the course of the r/DA reaction. According to the literature, the DA reaction between furfuryl alcohol and maleic anhydride adduct took place in a similar temperature range: DA at 50–60 °C (exothermic peak) and rDA at a temperature of 120–150 °C (endothermic peak) [21,35].

In the IGKF/T-UVECOAT3003 sample, in addition to the DA and rDA reactions, an endothermic deblocking process of polyisocyanate takes place in the range of 110–130 °C. The thermal effects of these processes overlap, and for this reason, the thermogram of this sample is flatter.

Under the influence of heating, in the powder coating sample L_HEMA/5MMA/2BA/IGKF/T/UVECOAT 3003, in addition to the DA, rDa, and deblocking reactions, a crosslinking process takes place between the deblocked isocyanate groups and the hydroxyl groups of the acrylic resin. This process is visible on the thermogram in the range of 120–150 °C as a broad exothermic peak.

**Figure 6:**
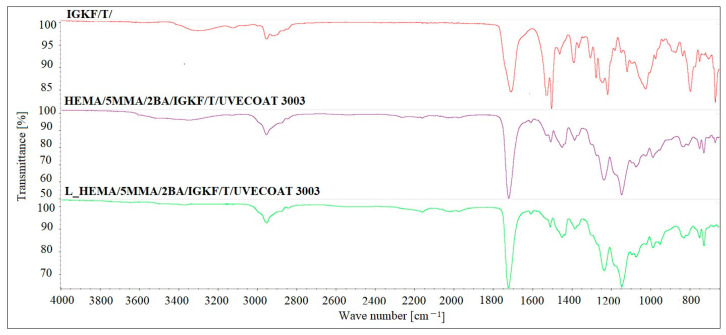
The FTIR spectra of IGKF/T/, L_HEMA/5MMA/2BA/IGKF/T/UVECOAT 3003 non-crosslinked and crosslinked powder coating.

The FT-IR analysis was used to confirm the blocking reaction of PICs and curing reaction of powder coatings (Figure 6). The FT-IR spectrum of bPICs showed no absorption in the range of 2250–2270 cm^−1^, which corresponds to the asymmetric C–N stretching vibration in the -NCO groups of diisocyanate. Moreover, no absorption at 3126 cm^−1^ originating from the N-H stretching vibration of triazole was observed. The absence of these bands indicates that the -NCO groups from the PICs were completely blocked by the triazole. The strong twin absorption bands in the range of 1500–1540 cm^−1^ in the spectrum of IGKF/T correspond to the C=N- aromatic stretching vibration of triazole [35].

The spectra of both non-crosslinked and crosslinked L_HEMA/5MMA/2BA/IGKF/T/UVECOAT 3003 are nearly identical, showing a urethane -NH stretching absorption at 3330 cm^−1^, a urethane -NH bending absorption at 1521 cm^−1^, and stretching vibrations of carbonyl groups (C=O) at 1700 cm^−1^. The urethane -NH bending absorption at 1521 cm^−1^ overlaps with the twin C=N- aromatic stretching vibration peaks derived from triazole. These twin peaks in powder coating spectra are weak because the IGKF/T content is low (12.8%). The intensity of these peaks in the spectrum of the crosslinked coating is slightly lower than in the non-crosslinked one, and this may indicate the partial evaporation of triazole from the coating during heating, which was confirmed by TGA analysis. This slight change in intensity indicates that an r/DA reaction has occurred. Otherwise, the intensity of this signal should decrease by at least half because there is only one C=N- bond in the DA adduct (Figure 4). In the range of 1600 cm^−1^, a weak signal is visible coming from the C=C stretching vibrations of the unsaturated UVECOAT 3003 resin. This signal is present in the spectrum of the crosslinked and non-crosslinked coating, which proves that the r/DA reaction has occurred and the C=C groups have been recreated and are capable of the DA reaction again.

The presence of polysiloxane is confirmed by Si-O-Si absorption in the 1020–1100 cm^−1^ range, while Si-CH_3_ shows absorption at 1220 cm^−1^ and 800 cm^−1^ [36]. The cured coatings confirm the progression of the reaction between the hydroxyl groups (-OH) of the acrylic resin and the isocyanate groups (-NCO) derived from the crosslinking agent within this range (3400–3600 cm^−1^). The characteristic C-O stretching vibrations are observed in the range of 1100–1200 cm^−1^. Absorption bands in the range of 2800–3000 cm^−1^ indicate the presence of aliphatic groups (-CH_2_, -CH_3_), while signals at 1411 cm^−1^ are characteristic of the C-H vibrations of methyl groups. Additionally, absorbance at 1230 cm^−1^ corresponds to the asymmetric stretching vibrations of C=O and O-CH_2_ bonds originating from the ester groups of the polyester resin [16].

In order to confirm the self-healing properties of the powder coating, polarized optical microscopy equipped with a heating table was employed. Before investigating the temperature required for healing the powder coating, a scratch was created under a load of 300 g. Subsequently, the temperature and time required for the self-healing of the coating were evaluated. It was observed that at 160 °C for 15 min, the crack was successfully stuck together. The results are presented in Table 2.

The powder coatings containing unsaturated polyester resin were characterized by self-healing properties. This means that under the influence of temperature, triazole with unsaturated resin created a DA adduct, allowing for the restoration of the powder coating.

**Figure 7:**
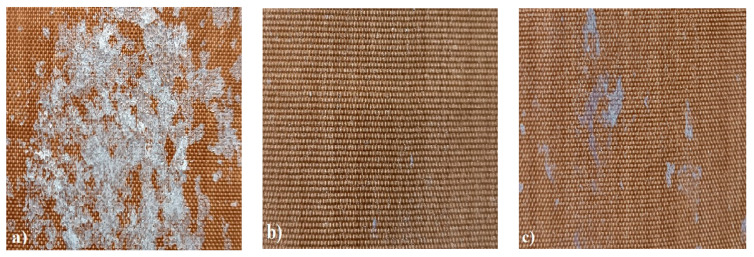
Image of sample of L_HEMA/5MMA/2BA/IGKF/T/UVECOAT 3003 (**a**) before healing, (**b**) after “healing”, and (**c**) after “healing” for the second time.

In order to test whether the coating is capable of repeated self-healing, the tests were performed by using polarized optical microscopy. The L_HEMA/5MMA/2BA/IGKF/T/UVECOAT 3003 coating was sprayed onto a Teflon tray and cured. Then, the created cured powder coating was cooled, cut into small pieces (Figure 7a), and heated again to 160 °C for 15 min. The powder coating stuck together (Figure 7b). When the operation was repeated a second time, the coating did not solidify completely (Figure 7c), which indicates a partial loss of self-healing ability.

**Figure 8:**
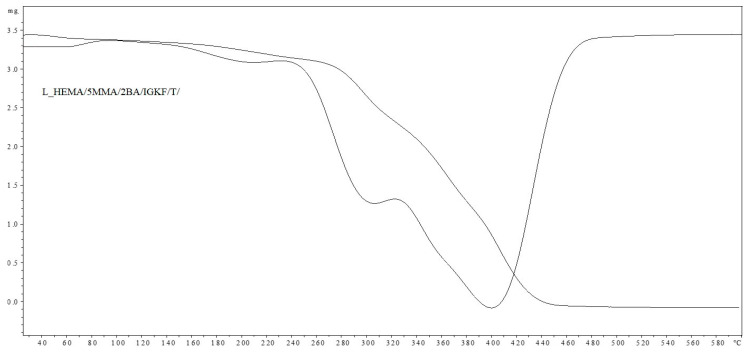
TGA and DTG curves of L_HEMA/5MMA/2BA/IGKF/T/.

**Figure 9:**
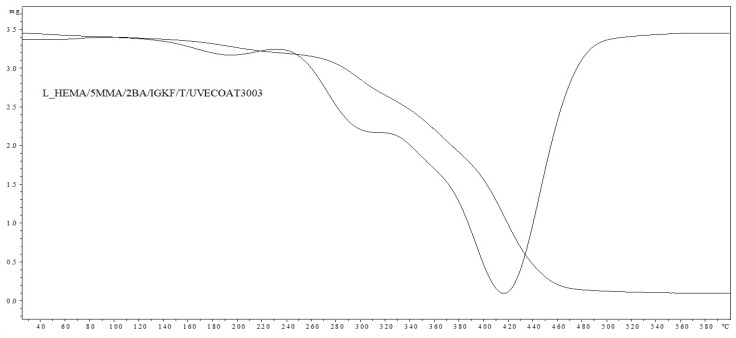
TGA and DTG curves of L_HEMA/5MMA/2BA/IGKF/T/UVECOAT 3003.

The observed partial loss of self-healing ability results from the partial release of 1,2,4-triazole (diene) from the coating, which was proven based on the results of the TGA analysis (Figure 8 and Figure 9). The weight loss at a temperature above 160 °C in the sample containing unsaturated polyester resin (dienophile) (L_HEMA/5MMA/2BA/IGKF/T/UVECOAT 3003) is lower (7.63%) than in the sample not containing the dienophile (10.54%), which indicates that some of the 1,2,4-triazole remains in the coating and takes part in the Diels–Alder reaction and some of it evaporates. This remaining amount of triazole in the coating is sufficient to ensure the complete self-healing of the coating during the first test, but during the next test, there may not be enough 1,2,4-triazole to ensure the complete self-healing of the coating.

The thermal stability of L_HEMA/5MMA/2BA/IGKF/T/ and L_HEMA/5MMA/2BA/IGKF/T/UVECOAT 3003 powder coatings was investigated with TGA and DTG analysis (Figure 8 and Figure 9).

In both cases, the coating compositions began to lose mass at temperatures above 160 °C. The mass loss in this range is 10.54% for the sample L_HEMA/5MMA/2BA/IGKF/T/ and 7.63% for L_HEMA/5MMA/2BA/IGKF/T/UVECOAT 3003. The maximum mass loss rate occurs at the temperature of Tmax1 = 195 °C for sample L_HEMA/5MMA/2BA/IGKF/T and at Tmax1 = 185 °C for sample L_HEMA/5MMA/2BA/IGKF/T/UVECOAT 3003, appropriately. This mass loss is related to the deblocking of triazole and the formation of polyisocyanate. The mass loss in this temperature range was slightly lower in the sample L_HEMA/5MMA/2BA/IGKF/T/UVECOAT 3003, which could be due to the reaction of triazoles with unsaturated resin UVECOAT 3003 and the formation of the DA adduct.

Furthermore, in the next step, the T_max2_ for L_HEMA/5MMA/2BA/IGKF/T/UVECOAT 3003 reaches 295 °C (with a weight loss of 34.72%). Meanwhile, this temperature for L_HEMA/5MMA/2BA/IGKF/T is 300 °C with a mass loss percentage of 63.92%. This step is related to the degradation of urethane bonds and triazole [36]. Slower weight loss in the range 240–380 °C for the L_HEMA/5MMA/2BA/IGKF/T/UVECOAT3003 coating suggests its higher thermal stability, which may be related to its higher density of crosslinking by DA adducts.

The third step (T_max3_ = 404 °C and T_max3_ = 418 °C for resin without and with UVECOAT 3003, respectively) is associated with the degradation of the acrylic and polyester resin as well as polysiloxane KF-6000 segments.

### 4.3. Characterization of Powder Coatings

The obtained powder coatings were also tested in terms of physical–mechanical properties. Table 3 lists the parameters for the investigated powder coatings.

A polymerization test was conducted to confirm the degree of crosslinking in the coating according to the technical requirements of Qualicoat [23].

No noticeable changes were observed after wiping the samples back and forth 30 times in each direction with a cotton swab soaked in methyl ethyl ketone (MEK), indicating the complete crosslinking of all samples. Comparing the reference powder coatings (L_HEMA/5MMA/2BA/IGKF/T/) with L_HEMA/5MMA/2BA/IGKF/T/UVECOAT3003 powder coatings, the sample containing unsaturated resin showed better hardness, scratch resistance, and adhesion.

Comparing the reference sample (L_HEMA/5MMA/2BA/IGKF/T/) with L_HEMA/5MMA/2BA/IGKF/T/UVECOAT3003 powder coatings, the coating containing unsaturated resin showed higher hardness, scratch resistance, and adhesion. Moreover, the L_HEMA/5MMA/2BA/IGKF/T/UVECOAT3003 coating showed much lower roughness and higher gloss than L_HEMA/5MMA/2BA/IGKF/T, which proves that the components have been correctly selected in terms of chemical structure because the improvement in these parameters shows their good compatibility. The water contact angle of the coating with the addition of unsaturated polyester resin is lower than that of the L_HEMA/5MMA/2BA/IGKF/T sample, which is a consequence of the higher hydrophilicity of the polyester resin compared to the acrylic resin. Column 3 of Table 3 contains the properties of the L_HEMA/5MMA/2BA/IGKF/T/UVECOAT3003 coating after the self-healing process, measured in the place where the scratch was made. However, the physico-mechanical properties at the self-healed site decreased even though visually the coating showed no significant differences. The gloss decreased slightly, and the roughness increased, but the mechanical properties deteriorated, in particular the adhesion to the substrate, cupping, and scratch resistance.

## 5. Conclusions

As a result of this research, a low-temperature, self-healing polyurethane powder coating was developed, which uses a releasing blocking agent in the self-healing process. In the case of classic polyurethane powder coatings, the blocking agent completely evaporates from the coating, but in our case, it was used in the self-healing process. The key is to select the blocking agent so that it has a diene moiety and at the same time is deblocked at a low temperature. In our research, 1,2,4-triazole used as a blocking agent worked well, and after deblocking, it underwent a Diels–Alder reaction with an unsaturated polyester resin and then, as a result of further heating, a retro-Diels–Alder reaction. However, a multiple self-healing test is not recommended because the coating did not heal completely after the second cooling cycle. The reason for this was the partial evaporation of triazole from the coating, which was confirmed by thermal analysis tests. The consequences of this were worse physical and mechanical parameters of the coating at the point of cracking after the self-healing process. However, these properties were better compared to the reference sample, which indicates that performing self-regeneration of the coating once is beneficial, but this process cannot be repeated many times due to the deterioration of the coating properties. The concept of this research opens up new opportunities to develop advanced polyurethane powder coatings with self-healing properties and limited blocking agent emissions. However, the blocking agent should be selected appropriately so that it is blocked at low temperatures and is difficult to evaporate from the coating; then, the self-healing process can be repeated more times.

## Figures and Tables

**Table 1:** Composition of the powder coatings.

Symbol of Powder Coating	HEMA/5MMA/2BA [%]	IGKF/T [%]	UVECOAT 3003 [%]
L_HEMA/5MMA/2BA/IGKF/T/	84.4	15.6	-
L_HEMA/5MMA/2BA/IGKF/T/UVECOAT 3003	69.6	12.8	17.6

**Table 2:** Examination and comparison of the self-healing ability of the cracks created on the powder coatings at room temperature and 160 °C.

Name of Sample	Room Temperature [23 °C]	Self-Healing Temperature [160 °C]
L_HEMA/5MMA/2BA/IGKF/T/	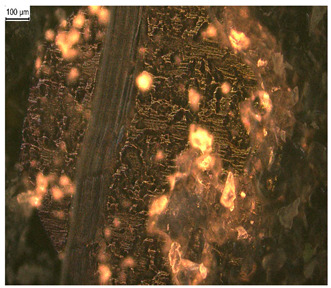	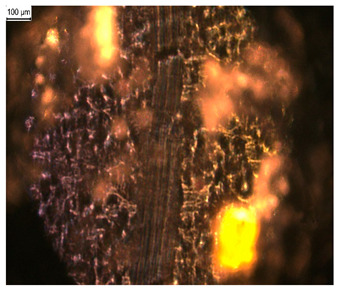
L_HEMA/5MMA/2BA/IGKF/T/UVECOAT 3003	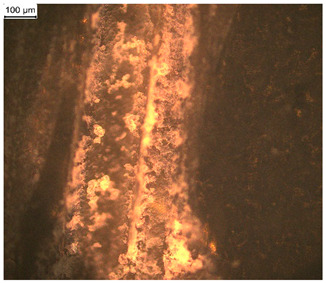	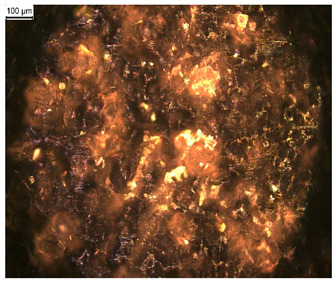

**Table 3:** Summary of physical–mechanical parameters of the powder coatings.

Symbol of Coatings	L_HEMA/5MMA/2BA/IGKF/T/	L_HEMA/5MMA/2BA/IGKF/T/ UVECOAT 3003 (After Curing)	L_HEMA/5MMA/2BA/IGKF/T/ UVECOAT 3003 (after Crack and Self-Healing Process)
Flowability [cm]	1.10	1.25	Not measured
Roughness: Ra/Rz	4.9/12.8	1.5/8.0	4.3/14.35
Gloss 60° [GU]	30.36	37.86	27.26
Thickness [um]	56.85	68.74	65.86
Relative hardness [-]	0.54	0.73	0.47
Adhesion to steel [0—good; 5—bad]	2	1	3
Scratch resistance [g]	300	800	250
Cupping [mm]	4.6	4.5	1.2
Water contact angle [deg]	107.23	84.13	82.19

## Data Availability

All data is contained within the article.
